# All the Right Noises: Background Variability Helps Early Word Learning

**DOI:** 10.1111/cogs.12539

**Published:** 2017-09-23

**Authors:** Katherine E. Twomey, Lizhi Ma, Gert Westermann

**Affiliations:** ^1^ Department of Psychology Lancaster University

**Keywords:** Word learning, Fast mapping, Variability, Entropy, Dynamic systems, Cognitive development

## Abstract

Variability is prevalent in early language acquisition, but, whether it supports or hinders learning is unclear; while target variability has been shown to facilitate word learning, variability in competitor items has been shown to make the task harder. Here, we tested whether background variability could boost learning in a referent selection task. Two groups of 2‐year‐old children saw arrays of one novel and two known objects on a screen, and they heard a novel or known label. Stimuli were identical across conditions, with the exception that in the *constant color* condition objects appeared on a uniform white background, and in the *variable color* condition backgrounds were different, uniform colors. At test, only children in the variable condition showed evidence of retaining label‐object associations. These data support findings from the adult memory literature, which suggest that variability supports learning by decontextualizing representations. We argue that these data are consistent with dynamic systems accounts of learning in which low‐level entropy adds sufficient noise to the developmental system to precipitate a change in behavior.

## 1 Introduction

Children's early word learning has long fascinated researchers. When a child hears the new word *spaceship*, linking it with a new toy flying machine rather than her toy dog—or indeed the flying machine's wings, its color, the way it moves, and so on—seems to pose little problem. Given that the space of potential referents is theoretically infinite (Quine, 1960), this ability to quickly map a novel word to a novel object is impressive; so impressive, in fact, that until recently children's ability to disambiguate has been attributed to metacognitive rules in the form of innate biases (Markman, 1994) or learned lexical constraints (Golinkoff, Mervis, & Hirsh‐Pasek, 1994). For example, given the choice between two familiar objects and a new one, children may reason that since the yellow thing is a banana, and the brown fluffy thing is their much‐loved toy dog, then the new word *spaceship* must refer to the new toy flying machine (mutual exclusivity or dysjunctive syllogism; Carey & Bartlett, 1978; Halberda, 2006; Markman & Wachtel, 1988; for other proposed constraints see Golinkoff, Hirsh‐Pasek, Bailey, & Wenger, 1992). Recent years have seen the emergence of a new view of word learning as a low‐level phenomenon that can proceed without invoking complex, metalinguistic awareness. These theories, often based on computational models, argue that low‐level associative processes of excitation and inhibition can give rise to the apparently complex behaviors children demonstrate during referent selection (RS; Horst, Samuelson, et al., 2011b; McMurray, Horst, & Samuelson, 2012; Samuelson, Kucker, & Spencer, 2016; Samuelson, Smith, Perry, & Spencer, 2011; Smith, 2000; Twomey, Morse, Cangelosi, & Horst, 2016).

While the debate as to the mechanisms underlying children's word learning goes on, it is clear that memory and language are linked from very early in development (Taylor, Liu, & Herbert, 2016; Zimmermann et al., 2015). In particular, learning a new word depends critically on children's ability to form and retain word‐object associations. There is mounting evidence that a single disambiguation event is not sufficient for full word learning; rather, children learn word‐object associations incrementally, forming in‐the‐moment mappings between labels and objects and strengthening memories of these mappings across repeated encounters via cross‐situational learning (McMurray et al., 2012; Smith & Yu, 2008; Yurovsky, Fricker, Yu, & Smith, 2014). Consequently, the field has recently focused on the multiple factors that affect early language acquisition, demonstrating that RS and word learning are flexible, even fragile processes which depend heavily on the temporal and visual availability of information in the learning environment, for example repetition, competition, and timing (e.g., Arias‐Trejo & Plunkett, 2010; Horst, Scott, & Pollard, 2010; Mather & Plunkett, 2009).

In line with the adult literature (Posner & Keele, 1968), developmental research has demonstrated that variability in target items is a key influencing factor in early learning. For example, visual variability encountered across target stimuli facilitates categorization in 6‐ to 7‐month‐old infants (Quinn & Bhatt, 2010), and phonological variability in affect or speaker has been shown to support early word recognition (Rost & McMurray, 2009; Singh, 2008). Recent work has shown that target variability also affects word learning: In an RS task, when shown a novel 3D object category with exemplars that varied in color, 30‐month‐old children learned category labels, but they did not when exemplars were identical or varied in shape and color simultaneously (Twomey, Ranson, & Horst, 2014; see also Ankowski, Vlach, & Sandhofer, 2013; Perry, Samuelson, Malloy, & Schiffer, 2010). Thus, while some target variability supports language learning, too much target variability appears to disrupt it. However, there are more sources of variability in the input to language learning than just the to‐be‐learned item. Findings from RS tasks in which variability is instantiated in competitor (i.e., non‐target) objects suggest that *lack* of variability supports word learning. For example, repeating competitor objects across RS trials, or reducing the number of competitor objects seen during RS, supports 2‐year‐old children's retention of label‐object associations (Axelsson & Horst, 2014; Horst et al., 2011b).

In addition, there is good theoretical reason to expect extraneous background variability—entropy—to support word learning. Specifically, evidence from adult problem‐solving studies suggests that introducing entropy to a task facilitates learning. For example, Stephen, Dixon, and Isenhower (2009) showed adults a series of gear system problems on a computer screen. Half of the participants saw the problems appear in a consistent spatial location, but for the other half, the task included added entropy in the form of random variability in stimulus location. While both groups eventually abstracted a short‐cut solution to the gear problems, the group for whom the task contained more entropy did so the fastest. The authors explained this finding in the context of dynamic systems theories of cognition and development. In these approaches, cognitive structure emerges from the dynamic interactions of multiple, coupled components including the learner's body, learning history, and in‐the‐moment characteristics of the task. Cognitive structure is instantiated as a stable state (*attractor*) in the behavior of this complex system. Dynamic systems of this type exhibit *phase shifts* from one attractor to another, resulting in qualitative and quantitative changes in the system's behavior. Because these phase shifts result in behavioral change, from the dynamic systems perspective, they reflect learning (Karmiloff‐Smith, 1992; Piaget, 1952; Thelen & Smith, 1996; for an explicit computational implementation of this theory, see Schöner, Spencer, & the DFT Research Group, 2015). As Stephen et al. (2009) demonstrate, extraneous entropy during learning destabilizes attractor states, speeding the onset of a phase shift; thus, non‐target variability should speed up early learning by helping new cognitive structure emerge via a shift from one stable state to another.

Despite this strong theoretical prediction, evidence for the effect of adding noise to early word learning is mixed. Background variability appears to boost performance in novel noun generalization tasks, which test children's ability to extend words heard in‐the‐moment to new category exemplars. For example, Goldenberg and Johnson (2015; see also Goldenberg & Sandhofer, 2013) trained 16‐ to 20‐month‐old infants with novel category exemplars on backgrounds which (a) repeated, (b) varied randomly, or (c) varied in a nonrandom presentation. Infants were then asked to generalize novel category labels to a new exemplar. For infants who saw variable but non‐random backgrounds, looking times during training predicted categorization ability. For infants who learned from repeated contexts or randomly varying backgrounds, however, there was no such relationship. Here, then, additional, structured context variability supported children's noun generalization.

While generalization tasks depend on online processing, typically of never‐before seen stimuli, RS tasks tap multiple timescales of behavior, including children's long‐term vocabulary (which scaffolds disambiguation) and in‐the‐moment task characteristics, as well as the child's ability to make links across these timescales (Kucker, McMurray, & Samuelson, 2015). In these tasks, which test children's memory of already encountered label‐object mappings after a period of time, background variability has been shown to hinder word learning. For example, teaching 3‐year‐old children novel words by reading them the same book every day over a week boosts their word learning relative to teaching the same novel words from multiple, different books (Horst, Parsons, & Bryan, 2011a; Williams & Horst, 2014). Thus, while the generalization literature predicts a beneficial effect of variability on retention, the storybook literature predicts a benefit of contextual consistency. Importantly, however, the prediction from dynamic systems theory (DST) that additional entropy should boost word learning in an RS task has yet to be explicitly tested.

This study addresses this gap. We added low‐level background variability to an RS paradigm on the hypothesis that variability should support retention of novel labels. Specifically, we gave 2‐year‐old children a looking time task in which trials were presented either on a white background or on multiple colored backgrounds. After a 5‐min break to allow for forgetting, children saw a single warm‐up trial followed by six retention test trials, in which the just‐seen novel objects were presented on a gray background for both groups of children. Based on dynamic systems accounts of learning, we expected children in the variable color condition to show stronger retention of label‐object associations than children in the constant color condition. Finally, we explored in detail the relationship between children's attention to novel targets during RS and their subsequent retention. Together, these results give a fine‐grained picture of children's looking behavior during RS and its relationship to subsequent word learning.

## 2 Method

### 2.1 Participants

Thirty typically developing, monolingual, English‐speaking, 2‐year‐old children (14 girls, *M *=* *22.77 months, *SD *=* *1.87 months; range* *=* *20.0–26.0 months) with a mean productive vocabulary of 176.04 words (*SD *=* *117.50 words, range* *=* *4–413 words) and no family history of colorblindness participated. Children were randomly assigned to the *constant color* condition (*n *=* *14) or the *variable color* condition (*n *=* *16). Children's ages and productive vocabularies did not differ between conditions (age: *t*(27.31)* *=* *0.83, *p *=* *.41; vocabulary: *t*(16.52)* *=* *1.07, *p *=* *.30). Data from six additional children were excluded from analyses due to fussiness as defined as crying/refusal to stay on caregiver's lap (1), parental labeling of target objects (3), bilingualism (1), and an eye tracker sample rate of under 25% (1). Parents were reimbursed for travel expenses and children received a storybook for participating.

### 2.2 Stimuli

**Figure 1:**
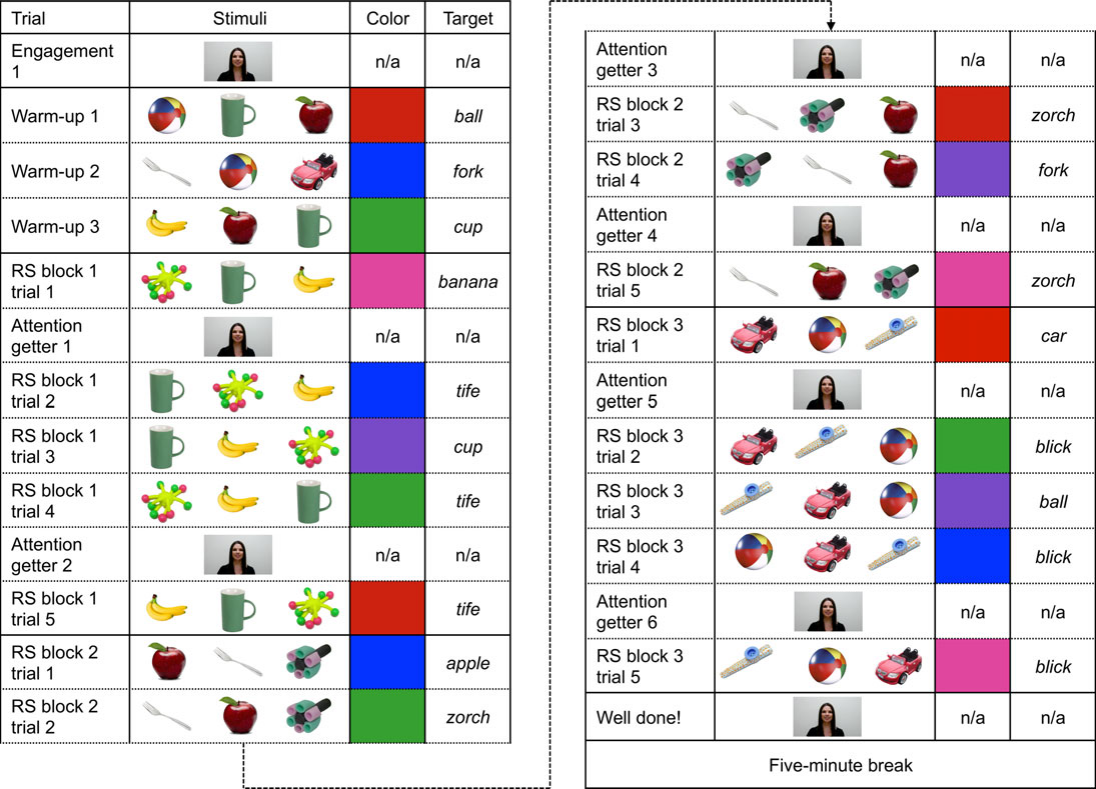
Example warm‐up and RS trial order, variable color condition. The corresponding trial order in the constant color condition was identical with the exception that the background color was always white. RS = referent selection.

**Figure 2:**
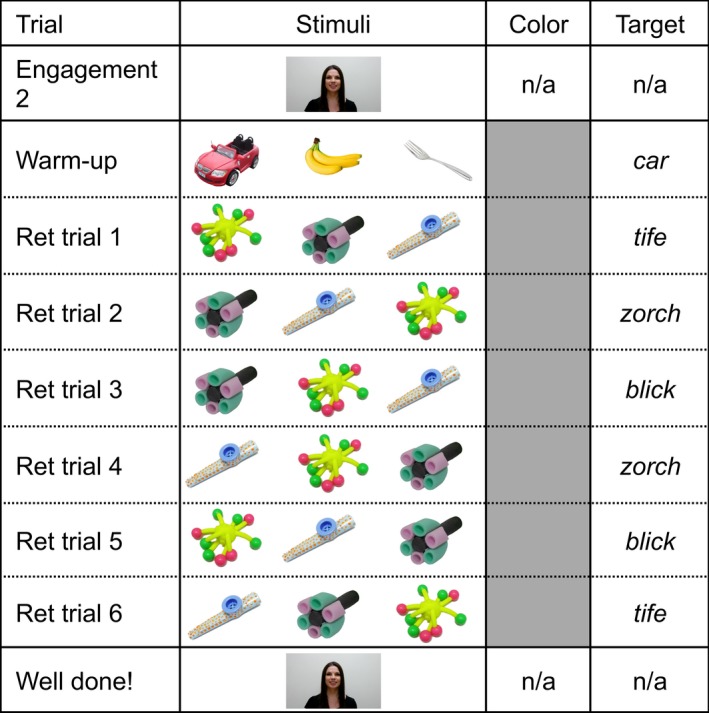
Example retention trial order. The retention phase was identical across conditions. Ret = retention.

The study consisted of three phases: warm‐up, RS, and retention (see Figs. 1 and 2). Critically, stimuli for each phase were identical across conditions with the exception that during warm‐up and RS, in the variable color condition objects appeared on colored backgrounds, and in the constant color condition backgrounds were always white. We also presented children with engagement and attention‐getting stimuli. We describe the details of stimuli for the different phases separately below. Overall, however, warm‐up, RS, and retention stimuli were videos containing 2D photographic images of known and/or novel objects (see Fig. 1). Known objects were an apple, a ball, a banana, a car, a cup, and a fork, and they were selected because their labels are familiar to children of this age group (Fenson et al., 1993). Novel objects were a purple, green, and black foam rocket (labeled *zorch*), a spherical yellow object with multiple flexible legs capped with pink and green balls (labeled *tife*), and a blue kazoo with raised orange spots (labeled *blick*), selected from an online database of objects unfamiliar to children of this age (NOUN Database; Horst & Hout, 2016). Each trial consisted of a single video of three objects. Videos were created in Microsoft Powerpoint 2010 and converted to AVI format, using Microsoft Windows Live Movie Maker 2011. Each video was accompanied by embedded audio consisting of the same female speaker saying, *Can you find the [label]? Look at the [label]! Where's the [label]?*, as well as sound effects to keep children engaged in the task. Known labels were the appropriate English labels for those objects, and novel labels were *blick* (kazoo), *tife* (legs/balls) and *zorch* (rocket), selected as plausible, but unfamiliar English object names. Auditory stimuli commenced 5 s after the start of each trial. First label onsets occurred from 0.78 to 0.90 s after the beginning of the auditory stimulus and offsets from 1.27 to 1.58 s; second label onsets from 2.20 to 2.48 s and offsets from 2.65 to 3.25 s; and third label onsets from 3.54 to 4.21 s and offsets from 4.22 to 5.19 s.

#### 2.2.1 Engagement stimuli

Engagement stimuli consisted of a 7 s video of a female experimenter on a white background, smiling and saying, *Hello! Let's play a game! Can you find what I'm looking for?* in child‐directed speech.

#### 2.2.2 Warm‐up stimuli

Warm‐up stimuli were 16 s videos, each depicting a set of three of the known objects and were designed to encourage children to look at the target object in response to its label. In the first 0.5 s, a small uniform color rectangle appeared in the center of a black screen and spun in a circle anticlockwise, growing as it did so until it filled the whole screen, at which point the rectangle served as the background on which the objects would appear. In the next 2 s, the three objects appeared in the top left‐hand corner of the screen and bounced diagonally downwards accompanied by a *boing* sound, coming to a rest in the center of the screen and remaining there for 9.5 s, during which time the target object was labeled three times (e.g., *Can you find the apple? Look at the apple! Where's the apple*?). In line with typical 3D object RS tasks in which warm‐up trials include ostensive feedback (e.g., Twomey et al., 2014), during the next 3 s, the target object rotated accompanied by a twinkling sound, followed by ostensive auditory feedback (e.g., *There's the apple!*). In the final 1 s, the objects bounced diagonally toward the bottom right‐hand corner and offscreen, accompanied by the sound of children cheering.

#### 2.2.3 Referent selection stimuli

Referent selection trials were 13 s long; they were identical to warm‐up trials with the exception that children saw one novel and two known stimuli, and there was no ostensive feedback phase. Background colors were either white (constant color) or pseudorandom (variable color), as in the warm‐up trials.

#### 2.2.4 Retention stimuli

Retention trials were 9.5 s; they were proceeded in an identical manner to RS trials except that the background was always gray and appeared immediately (i.e., there was no 0.5 s period where the background appeared) and all three objects were novel.

### 2.3 Procedure and design

Before the experiment began, the experimenter showed caregivers pictures of the known and novel objects to ensure they were appropriately known and novel to the child. All children were familiar with the known objects and unfamiliar with the novel objects. Caregivers were asked to complete a UK adaptation (Hamilton, Plunkett, & Schafer, 2000) of the MacArthur‐Bates Communicative Development Inventory (Fenson et al., 1994), a vocabulary index widely used to record receptive and productive vocabulary in children of this age. Caregivers completed the vocabulary index either before the experiment began or afterwards, depending on the child's level of engagement.

The eyetracking session took place in a quiet, dimly lit room. Children sat on their caregiver's lap 50–70 cm in front of a 21.5 in. 1,920 × 1,080 computer screen. A Tobii X120 eyetracker (Tobii Pro, Stockholm, Sweden) located beneath the screen recorded the child's gaze location at 17 ms intervals, and a video camera above the screen recorded the caregiver and child throughout the procedure. Caregivers were instructed not to interact with their child or look at the screen during the task to avoid biasing their child's behavior, and they were asked to sit at a 90° angle from their child to ensure the eyetracker tracked the child's eyes only.

The eyetracker was first calibrated, using a five‐point infant calibration procedure available in Tobii Studio. A small animation of a cartoon bird accompanied by a jingling sound appeared in each of the corners and the center of a 3 × 3 grid on a gray background while the eyetracker recorded infants’ gaze direction and duration. Calibration accuracy was checked for each child; recalibration was not necessary for any child. Immediately following calibration, children saw the engagement stimulus once.

#### 2.3.1 Warm‐up

The three warm‐up trials immediately followed the engagement stimulus. An example warm‐up phase for the variable color condition is depicted in Fig. 1. In the constant color condition, the background on each of the warm‐up trials was white. In the variable color condition, the background varied from trial to trial and was blue, green, pink, purple, or red. Which objects appeared, which served as targets, and object location were pseudorandomized across children such that no object appeared on more than two successive trials.

#### 2.3.2 Referent selection

Fifteen RS trials immediately followed the warm‐up phase. An example RS phase for the variable color condition is depicted in Fig. 1. Again, the corresponding warm‐up phase in the constant color condition was identical with the exception that backgrounds were white. RS trials were presented in three blocks of five trials for each set. Sets were kept constant across trials to maximize children's retention of novel labels (Axelsson & Horst, 2014); thus, one child might see a block of five repetitions of the *apple + fork + zorch* set, followed by the *banana + cup + tife* set, and finally the *car + ball + blick* set, with block order Latin square counterbalanced across children. In each RS block, children were asked to look at a known object on two trials and a novel object on three trials. Known/novel trial order and background color (variable color condition only) were pseudorandomized such that no more than two of the same trial type appeared in succession. Object location was also pseudorandomized.

During the RS phase, an attention‐getting stimulus appeared six times pseudorandomly such that it was always succeeded by at least one RS trial, and it consisted of a 3 s video of the speaker saying, *What's next?*. Finally, after the RS phase, children saw a 5 s video of the speaker saying, *Well done! All finished! See you soon!*

##### 2.3.2.1 Break

Following RS, children took a 5‐min break. During this time, they either remained on their caregiver's lap and watched an age‐appropriate animation or moved to a seating area in the same room and colored pictures from a book. In line with 3D object RS studies this break was designed to allow time for forgetting, to ensure the subsequent retention trials tested robustly learned associations (Horst & Samuelson, 2008).

##### 2.3.2.2 Warm‐up

A single warm‐up trial followed the break, in an identical manner to the previous warm‐up trials with the exception that objects were presented on a gray background. Recalibration in Tobii Studio is not required following the break, however post hoc calibration checks confirmed that calibration was equally accurate for retention and RS trials, with 85.04% and 85.46% of looks falling in one of the three areas of interest (AOIs) in each phase, respectively.

##### 2.3.2.3 Retention

Six retention trials immediately followed the warm‐up trial. Each object was labeled on two trials, and trial order and object location were pseudorandomized. Following the retention phase, children saw a 6 s video of the speaker saying, *Well done! It was fun to play with you! Thank you! Bye!*

### 2.4 Coding and data cleaning

Gaze position was calculated automatically in Tobii Studio (v. 3.2) by taking an average of the gaze position of both eyes during recording. Only timestamps for which the eyetracker reliably detected one or more eyes were retained (excluded: 88,770; 15.43%). Left, middle, and right AOIs were 350 pixels wide by 450 pixels tall and centered on each object's stationary position after they had bounced into the screen.

Non‐AOI looks were discarded, resulting in a final dataset of 309,586 RS and 61,247 retention gaze samples. Individual gaze samples were numerically coded (1 = target look, 0 = non‐target look), creating a raw looking time measure, which was further collapsed into 100 ms time bins. All subsequent analyses use this target looking measure, and they are standardized from the offset of the first label plus 233 ms (to account for saccade initiation latencies; Swingley, Pinto, & Fernald, 1999) to 6,733 ms post‐labeling.

## 3 Results

### 3.1 Referent selection

First, we were interested in whether background color variability affected children's looking on RS trials. We therefore calculated proportion target looking for each time bin as [time spent looking at target AOI/total AOI looking time]. We submitted this measure to a linear mixed effects model with fixed effects of time bin, trial type (novel, known), and condition (constant color, variable color) and their interactions, with random intercepts for participant and target item.^1^ Model output is provided in Table 1.

**Table 1:** Results from the linear mixed effects model

Effect	β	*SE*	*t*	χ^2^	*df*	*p*
**Time bin**	0.0022	0.00034	6.544	**163.80**	**1**	<.001***
Trial type	−0.019	0.070	−0.281	0.078	1	.78
Condition	−0.051	0.037	−1.366	0.59	1	.44
**Time bin × trial type**	0.00021	0.00056	0.368	**5.28**	**1**	.022*
Time bin × condition	0.00087	0.000478	1.855	0.084	1	.78
Trial type × condition	0.060	0.029	2.048	0.29	1	.59
**Time bin × trial type × condition**	−0.0020	0.00077	−2.65	**5.29**	**1**	.0081**

Note.

Values in bold were significant at the alpha = 0.05 level. * *p* < .05, ** *p* < .01, *** *p* < .001.

Overall, children's looking increased over time (positive main effect of time bin). This was modulated by an interaction between trial type and time bin, and further by a three‐way interaction between condition, trial type, and time bin. To explore these interactions further, we ran two separate mixed effects models on proportion target looking for each condition, each with fixed effects of time bin and trial type and random intercepts for participant and target item.

**Figure 3:**
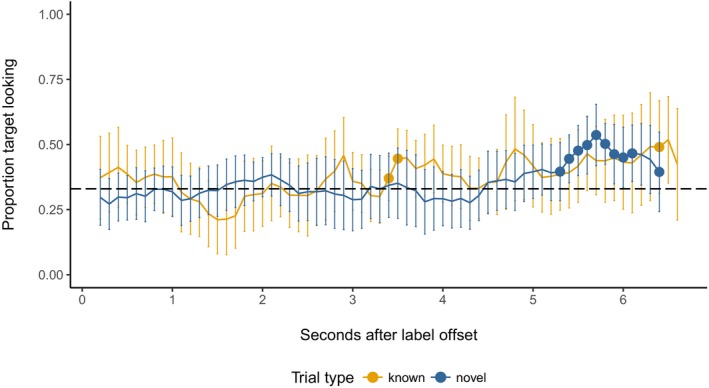
Proportion target looking in the constant color condition in 100 ms time bins. Error bars represent 95% confidence intervals. Where bins are marked with a point, looking is significantly above chance (0.33; *p* < .05, one‐sample, two‐tailed *t*‐test).

In the constant color condition, looking increased with time (main effect of time bin: β = 0.0022, *SE* = 0.00034, *t* = 6.55, χ^2^(1) = 73.41, *p *<* *.001). However, there was no effect of trial type (β = −0.018, *SE* = 0.083, *t *=* *−0.22, χ^2^(1) = 0.024, *p *=* *.88) or any interaction between time bin and trial type (β = 0.00022, *SE* = 0.00056, *t *=* *0.39, χ^2^(1) = 0.015, *p *=* *.70). To establish whether children correctly identified target items in response to labels, we plotted proportion target looking against time bin (Fig. 3). The *t*‐tests against chance revealed that although children demonstrated some above‐chance looking earlier in known trials (e.g., around 3,500 ms), sustained attention to the target for did not begin until around 5,500 ms, and then only on novel trials.

**Figure 4:**
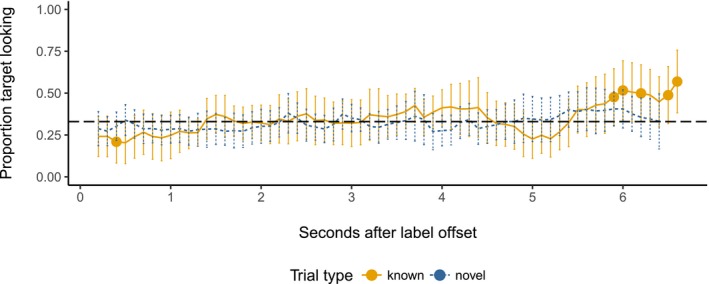
Proportion target looking in the variable color condition in 100 ms time bins. Error bars represent 95% confidence intervals. Where bins are marked with a point, looking is significantly above chance (0.33; *p* < .05, one‐sample, two‐tailed t‐test).

In the variable color condition, target looking also increased with time (β = 0.0031, *SE* = 0.00032, *t *=* *9.64, χ^2^(1) = 89.67, *p *<* *.001). While there was no independent main effect of trial type (β = 0.042, *SE* = 0.054, *t* = 0.77, χ^2^(1) = 0.16, *p *=* *.69), trial type did interact with time bin (β = −0.0018, *SE* = 0.00052, *t* = −3.51, *χ* ^2^(1) = 12.29, *p *=* *.00046). Again, to explore the interaction, we ran further separate mixed effects models on the variable color data for each trial type, with a main effect of time bin and random intercepts for participant and target type. These models confirmed that although target looking increased overall on both known and novel RS trials for children in the variable color condition, it did so faster on known than on novel trials (main effect of time bin; known: β = 0.0031, *SE* = 0.00035, *t *=* *8.78, χ^2^(1) = 76.41, *p *<* *.001; novel: β = 0.0013, *SE* = 0.00030, *t* = 4.39, χ^2^(1) = 19.17, *p *<* *.001). Finally, we plotted proportion target looking per time bin. As Fig. 4 shows, target looking reaches above‐chance levels on known trials, but not on novel trials.

Overall, then, children's looking to the target increased during RS trials. However, this effect was reduced when children in the variable color condition were asked to identify novel targets. Only children in the constant color condition showed sustained attention to the correct referent in response to known and novel words.

### 3.2 Retention

During RS, background color variability affected children's performance such that while children in the constant color condition showed some evidence of sustained attention to the correct novel referent, in the variable color condition children's target looking increased more quickly in response to known labels, although target looking was at chance overall.

**Figure 5:**
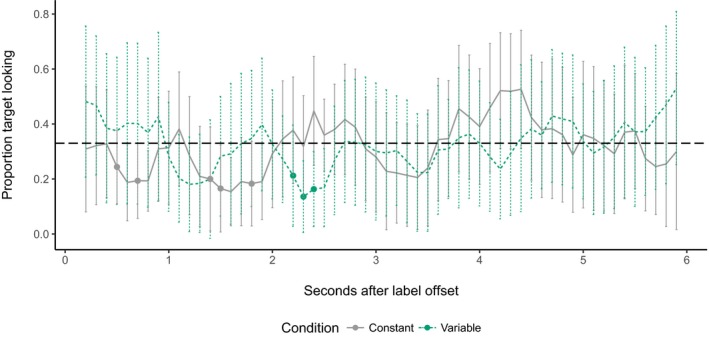
Proportion target looking during Block 1 of retention in 100 ms time bins. Error bars represent 95% confidence intervals. Where bins are marked with a point, looking is significantly above chance (0.33; *p* < .05, one‐sample, two‐tailed *t*‐test).

Next, we divided the retention trials in two blocks (i.e., Trials 1–3 vs. Trials 4–6), in line with existing work, which shows that providing children with a brief reminder of previously learned stimuli boosts test performance (e.g., Morgan & Hayne, 2006). Fig. 5 depicts looking times during Block 1 and shows little difference in target looking between the two conditions. This conclusion was supported by a mixed effects model with main effects of time bin and condition and their interaction, with by‐participant and by‐item random intercepts. As in RS, there was a small but robust increase in looking with time (β = 0.0019, *SE* = 0.00063, *t *=* *2.99, χ^2^(1) = 10.49, *p *=* *.0012). However, condition had no independent effect on looking times, and did not interact with time bin (main effect of condition: β = 0.043, *SE* = 0.00098, *t *=* *0.66, χ^2^(1) = 0.12, *p *=* *.73; time bin × condition interaction: β = −0.0080, *SE* = 0.00098, *t *=* *−0.81, χ^2^(1) = 0.67, *p *=* *.41).

**Figure 6:**
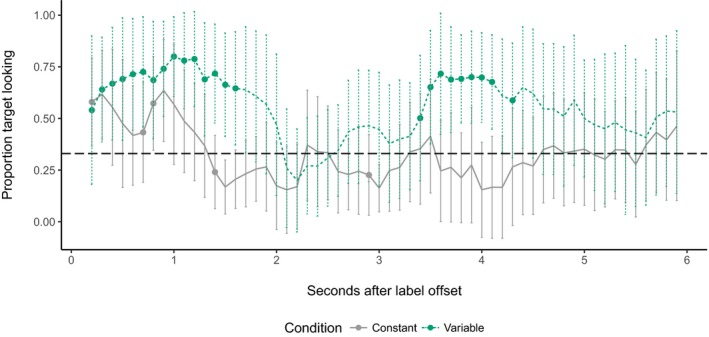
Proportion target looking during Block 2 of retention in 100 ms time bins. Error bars represent 95% confidence intervals. Where bins are marked with a point, looking is significantly above chance (0.33; *p* < .05, one‐sample, two‐tailed *t*‐test).

Data from Block 2 show a markedly different pattern, however. As Fig. 6 illustrates, children in the variable color condition robustly looked at the target at above‐chance levels immediately following labeling and again at around 4,000 ms, suggesting that encountering variable colored backgrounds during RS facilitated their retention of the novel label‐object mappings. A mixed effects model with the same fixed effects structure as above and by‐participant and by‐item random intercepts and slopes for condition revealed that on these later trials children's proportion target looking decreased over time (main effect of condition: β = −0.0029, *SE* = 0.00078, *t *=* *−3.65, χ^2^(1) = 32.09, *p *<* *.001). This effect was constant for children in either condition (time bin × condition interaction: β = −0.00084, *SE* = 0.0012, *t* = −0.71, χ^2^(1) = 0.52, *p *=* *.47). Critically, however, proportion target looking was greater for children in the variable color condition than in the constant color condition (main effect of condition: β = 0.25, *SE* = 0.078, *t *=* *3.26, χ^2^(1) = 9.38, *p *=* *.0022).

### 3.3 The relationship between RS and retention

Clearly, only children in the variable color condition retained label‐object mappings in Block 2; however, these children did not look at targets at above‐chance levels during RS. We reasoned that despite this apparent failure during RS, children must have learned something in order to show retention. If so, we expected a relationship between looking times during RS and children's retention. To test for this possibility, we calculated children's raw looking to both known and novel targets for the entirety of each RS trial. We then correlated these raw looking time scores with children's post‐label target looking during all six retention trials, excluding RS data from two children who looked away during the retention phase (variable: 2). In neither condition did we find a relationship between known target looking and retention (constant: *r*(12) = .50, *p *=* *.071, 95% CI [−0.05, 0.80]; variable: *r*(12) = .20, *p *=* *.50, 95% CI [−0.37, 0.65]). However, there was a positive correlation between novel target looking and retention, again in both conditions (constant: *r*(12) = .64, *p *=* *.014, 95% CI [0.16, 0.87]; variable: *r*(12) = .74, *p *=* *.0024, 95% CI [0.34, 0.91]). Thus, although children did not consistently direct their attention to targets during RS, they were nonetheless learning, with a stronger relationship between looking time and retention in the variable color condition.

### 3.4 Characterizing looking during RS

Although the correlations do not establish a causal link between looking times during RS and retention, they do suggest that children were learning during RS despite apparently not looking at target objects at levels greater than expected by chance. We therefore conducted a series of exploratory analyses to shed light on this unexpected finding. A possible explanation for these results is that some children were not responding correctly. If some—but not all—children looked consistently at competitor objects in response to the label instead of the target, overall looking would be at chance levels. Alternatively, infants may have been comparing stimuli (Kovack‐Lesh, Horst, & Oakes, 2008; Kovack‐Lesh, Oakes, & McMurray, 2012) or disambiguating (Halberda, 2006; Horst et al., 2010) during RS; in this case we would expect to see switching between stimuli on these trials. We therefore calculated the number of post‐labeling stimulus‐to‐stimulus transitions (Gabadinho, Ritschard, Mueller, & Studer, 2011) for each child on each RS trial, ignoring background looking and looks away (note that including transitions between stimuli and background would increase the overall number of transitions; this is therefore the most conservative approach to the following analysis).

**Figure 7:**
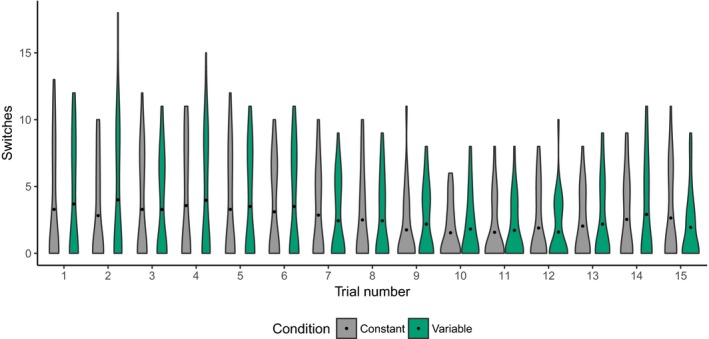
Switches between stimuli during referent selection. Bar width represents probability density. Black points represent mean switches.

Fig. 7 depicts switching post‐labeling for each trial. If, as a group, children were responding by attending to a single object (whether target or competitor), switching rates should be consistent and close to 1. In fact, the height of the bars indicates that children varied in their switching behavior; further, mean switches were higher than 1. Interestingly, across trials, the probability of low switching rates increased, as indicated by shorter bars with wider bases—this is the pattern we would expect if children were learning during RS. On the other hand, the more variable responding earlier in RS indicates higher rates of switching.

**Figure 8:**
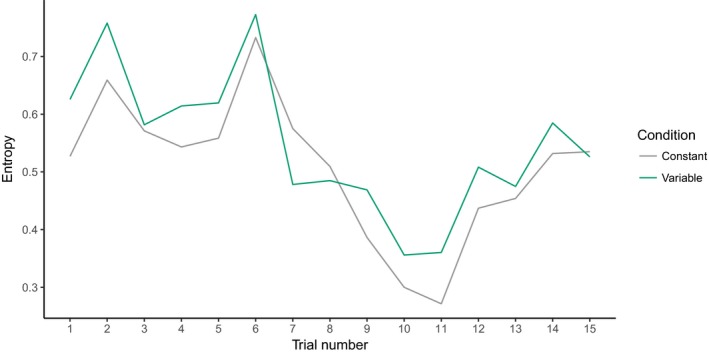
Mean entropy scores across referent selection trials.

Next, we quantified the variability in children's switching by calculating a Shannon entropy score (Gabadinho et al., 2011) for the sequences of looks generated by each child on each trial. Mean entropy scores per trial are depicted in Fig. 8. On a given trial, reliable looking to a single object would yield a Shannon entropy score of 0, while looking distributed evenly across stimuli would yield a score of 1. Clearly, looking behavior during RS is not consistent; rather, it is characterized by switching between stimuli. Entropy drops around trials 10 and 11, suggesting that children were responding more reliably toward the end of RS—again consistent with learning across trials.

### 3.5 A relationship between switching during RS and retention?

We then asked whether switching rates related to retention, based on findings from the categorization literature with infants demonstrating that infants who switched frequently between stimuli showed better categorization (Kovack‐Lesh et al., 2012). For this analysis, we included all switches made during both the pre‐ and post‐labeling phase, assuming that children could learn about the objects on screen before the label was heard. Overall switching rates did not differ between conditions (constant: *M *=* *146.64, *SD* = 41.91, range = 36–215; variable: *M *=* *151.75, *SD* = 36.06, range = 39–204; *t*(25.84) = −0.35, *p *=* *.73, 95% CI [−34.66, 24.45]). Because children with overall longer looking times had more opportunity to switch, we performed partial correlations on total switching during RS and retention scores (post‐label target looking times during retention), controlling for total looking times to all objects during RS. We removed data from two children whose total number of switches was more than two standard deviations outside the overall mean (constant: 1; variable: 1). These tests revealed no relationship between switching and retention scores either overall (*r*(28) = −.33, *p *=* *.09, 95% CI [−0.63, 0.057]) or separately by condition (constant: *r*(12) = −.46, *p *=* *.13, 95% CI [−0.81, 0.15]; variable: *r*(14) = −.39, *p *=* *.16, 95% CI [−0.73, 0.24]). Interestingly, then, we found no influence of background variability on switching rates as might be expected if infants in the variable condition were more distracted, for example.

### 3.6 Block effects and switching in retention

The final question posed by these data is why children in the variable color condition did not show retention until Block 2 of the test trials. One possibility is that switching between stimuli on Block 1 could have reactivated previously learned mappings, allowing them to respond correctly to the label on Block 2; if so, as in RS, looking times during Block 1 could be related to retention on Block 2. Thus, we correlated raw target looking times during the entirety of Block 1 with retention on Block 2 as indexed by post‐label target looking, removing data from three children who looked away for one or both blocks (constant: 1; variable: 2). Looking times on Block 1 were related to retention in the variable condition only (constant: *r*(11) = .37, *p *=* *.22, 95% CI [−0.23, 0.76]; variable: *r*(12) = .64, *p *=* *.014, 95% CI [0.16, 0.87]), reflecting our earlier finding that novel target looking during RS was related to retention for these children. Although there was no relationship between switching during RS and retention on Block 2, it remains possible that this relationship could hold for switching on Block 1 and retention. We therefore calculated total switching during retention for all children on Block 1 and correlated this with retention scores on Block 2, controlling for total looking times during Block 1. No relationship between switching and retention was found, either overall (*r*(28) = −.18, *p *=* *.38, 95% CI [−0.52, 0.21]) or for each condition individually (constant: *r*(14) = .19, *p *=* *.53, 95% CI [−0.40, 0.67]; variable: *r*(14) = −.20, *p *=* *.51, 95% CI [−0.68, 0.39]). As in RS, then, while we found no evidence that the amount of switching between stimuli related to retention, the amount of looking time accumulated *as a consequence* of switching was related to retention. Overall, these exploratory analyses of children's raw looking time data suggest that “chance” responding is a statistical artifact of more complex exploratory behavior which can lead to learning without prolonged target fixation (cf., Kovack‐Lesh et al., 2012).

## 4 Discussion

This study explored whether extraneous background variability would boost young children's word learning. We trained two groups of 2‐year‐old children with novel label‐object associations via multiple RS trials. Stimuli presented to both groups were identical except that during RS half the children saw arrays of novel objects displayed on a white background each time (constant color condition), and half saw objects on multiple colored backgrounds (variable color condition). During RS, children in both conditions increased their looking to target objects across trials but showed little evidence of sustained target looking at levels greater than expected by chance. Overall, then, the effect of background variability during this learning phase was weak. In contrast, at test there was a clear effect of background variability: In the second block of retention trials, children who had seen variable backgrounds during RS looked for longer at target objects than did children who had seen constant colored backgrounds, and they did so at levels greater than would be expected by chance. Thus, infants who had seen objects on variable backgrounds learned and retained the novel object‐label mappings, but infants who had seen the objects on a constant background did not. These results indicate that background variability facilitated word learning, raising several interesting issues.

### 4.1 How does “poor” performance during RS lead to retention?

The variable color condition is one of the first word learning experiments to show children performing apparently poorly during RS but successfully at test. In typical analyses of word learning tasks with 3D objects, test trials for which children have not correctly mapped novel labels during RS are excluded, with the rationale that children do not learn (correct) novel label‐novel object mappings when (incorrectly) mapping novel labels to known objects (e.g., Hilton & Westermann, 2016; Twomey et al., 2014). From this perspective, it is surprising that children in the variable color condition were the only ones to show robust retention: If during training these children were not looking to novel objects at above‐chance levels, how then did they retain?

Importantly, the canonical measure of attention on word learning tasks is proportion target looking out of all AOI looking, and it was on this measure that children seemed to be unsuccessful during RS and should therefore not be learning label‐object mappings. In contrast, our correlation analyses of raw looking times showed that children in the variable condition who spent longer looking at novel targets during RS (and the first block of retention) did indeed show better learning (for a similar result in a noun generalization task, see Goldenberg & Johnson, 2015). Then, our exploratory analyses revealed that this learning occurred while children were distributing their attention between targets and competitors (see also Fitneva & Christiansen, 2011). In particular, incorrect but reliable responding would lead to low entropy in the sequences of children's switching between stimuli, whereas entropy during RS was initially high for both known and novel trials. This finding is convergent with new empirical and computational evidence suggesting that word learning is a multiple‐stage process which entails (a) learning about non‐target as well as target objects and (b) encoding new objects as well as new word forms (Bion, Borovsky, & Fernald, 2013; Halberda, 2006; Horst et al., 2010; McMurray, Horst, Samuelson et al., 2011b; Yurovsky et al., 2014). These incremental accounts of word learning also suggest that low levels of looking during RS may be sufficient for children to demonstrate retention (cf., Yurovsky et al., 2014)—and this is what we found.

Thus, children in this study learned by accumulating experience with objects by switching between them. Importantly, however, it was the amount of experience gained rather than the number of switches that related to retention. This finding contrasts with work in the categorization literature, which demonstrates that the frequency of switching between stimuli is related to category learning. For example, Kovack‐Lesh et al. (2012) demonstrated that 4‐month‐old infants showed better category learning when they had switched more frequently between stimuli during familiarization (see also Kovack‐Lesh, McMurray, & Oakes, 2014; Kovack‐Lesh et al., 2008). However, there are many differences between these studies and the current work: The children who took part in the categorization studies were much younger, stimuli were displayed individually on two separate monitors rather than side by side on a single screen, and there was no labeling, to name a few. Thus, we interpret the lack of relationship between switching and learning in this study as inconclusive. Rather, our results suggest that the fine‐grained temporal dynamics of children's looking in word learning task is a rich topic for further investigation.

Finally, there remains the question of why the correlations between raw looking times during RS and retention only hold for the variable color condition. While it is impossible to draw firm conclusions from the null results in the constant color condition, we believe that the most likely explanation for this is a floor effect. Specifically, children in the color condition may have been learning during RS, but some other aspect of the task may have prevented them from responding correctly during retention. We discuss a possible mechanism below.

This study makes the empirical point that even if children are not responding at above‐chance levels during RS on proportion‐based analyses, they can learn enough about novel labels and novel objects to form partial label‐object mappings sufficient to support correct responses at test. This finding has implications for future work in word learning and, more broadly, developmental work involving forced‐choice tasks. First, when analyzing rich eye tracking data, proportion target looking may not be the only informative measure of children's performance: Analyses of raw looking times may reveal additional patterns in the data. Second, we provide strong evidence that children learn something about label‐object mappings even when they do not show a clear label response. Thus, researchers should consider including in their analyses test trials for which children did not correctly map novel labels during earlier training trials—even in 3D object paradigms—because children may well learn something from these trials even when making an erroneous mapping. Third, tasks involving a pointing response require infants to make an explicit choice via a point or a reach, resulting in substantially less noisy scores than seen in looking time measures (Ambridge & Lieven, 2011). In contrast, infants’ and children's looking times in general, and label responding in particular, are noisy and highly sensitive to individual differences in processing speed (Fernald, Perfors, & Marchman, 2006; Marchman & Fernald, 2008). These individual differences may have compounded children's apparently poor RS performance in this study, given that children of the same age in 3D object studies have repeatedly been shown to succeed at this type of task (e.g., Axelsson, Churchley, & Horst, 2012; Horst & Samuelson, 2008; Horst et al., 2010; Kucker & Samuelson, 2012; Twomey et al., 2014). Thus, this work underscores the importance of understanding the relationship between the explicit measures obtained from children's behavioral responses and the implicit measures provided by eye tracking, both in early language acquisition tasks and in developmental science more broadly (cf., Macdonald, Brandt, Theakston, Lieven & Serratrice, 2017; Noble, Rowland, & Pine, 2011). Future work is planned to explore this critical methodological issue.

### 4.2 Memory reactivation in word learning tasks?

Children in the variable color condition looked at target objects at chance levels on the first block of retention trials, but they responded systematically to labels on the second block. This behavior is consistent with the infant and adult memory literatures, which demonstrate that memory recall is boosted when participants are reminded of the previously studied materials before test (Hildreth & Rovee‐Collier, 1999; Hsu, Rovee‐Collier, Hill, Grodkiewicz, & Joh, 2005; Rovee‐Collier, Sullivan, Enright, Lucas, & Fagen, 1980; Smith & Handy, 2014). This phenomenon has been demonstrated in visual recognition studies in infants of this age group, for whom a 1 s visual reminder of previously learned stimuli is sufficient to trigger retention effects for novel stimuli (Morgan & Hayne, 2006).

Our exploratory analyses offer a mechanism for this effect: In Block 1, children explored stimuli without showing systematic responses to the label, but in doing so they reactivated their memory for the label‐object mappings learned during RS, which allowed them to demonstrate retention in Block 2. Often in word learning studies children are provided with only a single retention trial for each object, but the current data suggest that null findings could be due to difficulty in recall rather than a lack of learning. Future work is necessary to explore in detail whether the inclusion of memory reactivation‐type trials in retention tasks will provide a useful means of teasing apart “true” lack of retention from lack of responding. Moreover, whether the relationship exists between this phenomenon as seen in our data and other word learning paradigms such as the 3D object task is unknown. For example, Horst and Samuelson (2008) analyzed retention by block and found no such reactivation effect. Thus, establishing the locus of the effect of memory reactivation in the word learning field is critical for a thorough understanding of the delicate memory processes underlying early language acquisition.

### 4.3 Decontextualization in children's representational development

The fact that children in the constant color condition failed to retain newly formed label‐object associations is surprising. Indeed, related work indicates that consistency in context *supports* learning in related tasks in children of this age (e.g., Horst, Samuelson, et al., 2011b; Williams & Horst, 2014)—the opposite of the current findings. For example, Vlach and Sandhofer (2011) demonstrated that 2.5‐year‐old children's noun generalizations were supported by matching training and test contexts (i.e., same color), while only older children were able to generalize after training with variable contexts. Similarly, Axelsson and Horst (2014) showed that contextual consistency in the form of repeating rather than varying competitor objects supported children's word learning in an RS task similar to the one presented here. These studies suggest that simplification of non‐target information helps children learn about the target. From this perspective, children in the constant color condition should show better, not worse, learning than children in the variable color condition.

However, this is not what we found. Why, then, should a simpler task (i.e., constant color vs. variable color backgrounds) lead to worse learning? In fact, our results are in line with a wealth of adult memory work that demonstrates that background variability supports recall of learned information in a new context (e.g., Gartman & Johnson, 1972; Godden & Baddeley, 1975, 1980). More recent work has explored the effect of context on adults’ category learning. For example, Smith and Handy (2014) demonstrated that encountering faces on variable video backgrounds facilitated retention of names learned for those faces, while Finch, Carvalho, and Goldstone (2016) showed that variable backgrounds led to better retention of previously seen exemplars of a bird category.

These results are attributed to a *decontextualization* mechanism. When memories are formed after a single encounter, the context as well as the target is encoded. On subsequent encounters, if the context is unchanged, it remains part of the representation. For these context‐dependent memories, recall is impaired when the context changes. Godden and Baddeley (1975) describe a classic example of this context effect on memory, showing that divers who had learned word lists either on dry land or underwater were better at recalling words learned underwater when tested underwater, and better at recalling words learned on dry land when tested on dry land. When an item is encountered in multiple different environments, however, the representation becomes decontextualized: The context becomes less important to the representation, increasing the signal‐to‐noise ratio (Hintzman, 1984). If an item with a decontextualized representation is encountered in a new environment, then, it is easier to recall than if the representation were context dependent.

The same mechanisms that explain these adult data can account for children's word learning in this study. During RS, children in the constant color condition learned context‐dependent representations in which label‐object mappings were only associated with a white background. In contrast, children in the variable color condition learned decontextualized representations, where label‐object mappings were associated with multiple different backgrounds. At test, children encountered novel objects on a gray screen—and critically, neither group had seen these objects in this context until this point. Thus, any correlation between novel target looking during RS and retention for children in the constant color condition was likely washed out by the difficulty for these children of generalizing novel label‐object mappings to a new context *for the first time* (for a related argument, see Goldenberg & Johnson, 2015).

This raises the question of why contextual consistency in previous studies supports word learning. It is possible that different types of context have qualitatively different effects. In the current RS task, in line with Stephen et al. (2009), “context” was low‐level background variability (cf., Goldenberg & Johnson, 2015; Goldenberg & Sandhofer, 2013). In contrast, the contexts in the storybook literature are rich and salient: In these studies, books were constructed from photograph‐like images, resulting in a complex visual scene that varied from page to page. In addition, the sentence contexts in which novel words appeared also varied (Horst, Samuelson, et al.,2011b; Williams & Horst, 2014). Similarly, in the RS work, “context” consisted of the competitor objects presented alongside the targets, which were considerably more complex than a simple block of color (Axelsson & Horst, 2014). Thus, it may be that in rich learning environments, restricting complexity supports learning (e.g., Radesky & Christakis, 2016), while in simpler learning environments, increasing complexity by adding low‐level background noise helps learning. Clearly, the current data indicate that the relationship between RS and retention is not as straightforward as accurate responding during learning leading to accurate recall, and it deserves more in‐depth work (for a similar argument, see Samuelson et al., 2016).

### 4.4 Relationship to dynamic systems accounts of learning

Decontextualization of memory traces can account for the current data. However, we view this explanation as part of a much broader, more complex mechanism which can account for learning and development across multiple domains: dynamic systems, and in particular, the DST of development (Thelen & Smith, 1996). In DST, behavior emerges as a stable state of a complex system of multiple interacting components, and development—learning—is the transition between these stable states. Importantly, the developing child is not a closed system: S/he is embedded in a system consisting of myriad and constantly changing inputs, for example, the learning environment, the child's learning history, the child's own body, and subtle changes in the task at hand (e.g., Clerkin, Hart, Rehg, Yu, & Smith, 2017; Fausey, Jayaraman, & Smith, 2016; Morse, Benitez, Belpaeme, Cangelosi, & Smith, 2015; Samuelson & Horst, 2007; for a review, see Schöner et al., 2015). Changes to any of these components can have cascading and often unpredictable effects on the interactions at work in the learning system. Here, we tested the broad theoretical prediction from the dynamic systems approach that adding background entropy to the learning system should facilitate learning by speeding up the emergence of new stable behavioral states (Dixon, Stephen, Boncoddo, & Anastas, 2010; Stephen & Dixon, 2009). The current data support this account: Only the children who were trained in a higher entropy environment showed evidence of learning. Thus, decontextualization may serve as a mechanistic description of a phenomenon emerging from a higher order dynamic system.

Importantly, visual variability may be only part of the story: Other types of entropy may also support learning, raising the intriguing possibility for future work that entropy introduced in a different modality, for example sound or spatial location, could also support word learning. Further, this experimental work motivates further naturalistic studies to explore the effect of entropy in early language learning in the noisy, unconstrained real‐world environment encountered by children outside the lab (e.g., Clerkin et al., 2017). Overall, however, the current work provides evidence that word learning is just one component of a developmental system; critically, then, to understand language development, we must endeavor to understand the interactions of the components of the system as a whole.

## Note

1 Mixed effects models were run in the R package lme4.0 (v.1.1‐12; Bates, Mächler, Bolker, & Walker, 2015) in R Studio 0.99.903 (R Studio Team, 2015) running R version 3.2.4 (R Core Team, 2016). All random effects structures reported were the maximal that converged (Barr, Levy, Scheepers, & Tily, 2013), and all *p*‐values associated with mixed effects models were obtained, using the likelihood ratio test (anova()) and Type I sequential sum of squares.
